# Supplementary material for: COVID-19 Clinical Profiles and Fatality Rates in Hospitalized Patients Reveal Case Aggravation and Selective Co-Infection by Limited Gram-Negative Bacteria
Source: Int J Environ Res Public Health. 2022 Apr 26;19(9):5270. doi: 10.3390/ijerph19095270 (PMC9101447; doi:10.3390/ijerph19095270)
Supplement: Supplementary file 1 [file ijerph-19-05270-s001.zip › ijerph-1656560-supplementary.pdf]

Supplementary Material Table S1 . COVID-19 Clinical profiles, bacterial coinfection, and patient demographics and outcomes in Ha'il, KSA

No	bacteria	Specimen*	age +	Intubated?	h O2	LALC	Death
1	*K.pneumoniae*	Bloody,sputu	66	yes	vent	4.5	Yes
2	*K.pneumoniae*	Bood,urine	52	yes	vent	4.7	Yes
3	*K.pneumoniae*	Blood,urine	73	yes	vent	4.9	Yes
4	*K.pneumoniae*	Sputum	55	yes	vent	4.9	Yes
5	*K.pneumoniae*	Sputum	65	yes	vent	4.6	Yes
6	*K.pneumoniae*	Blood	68	yes	vent	5.5	Yes
7	*K.pneumoniae*	sputum	85	yes	vent	4.9	Yes
8	*K.pneumoniae*	Blood-a	38	yes	vent	4.7	
9	*K.pneumoniae*	Blood-an		yes	vent	4.9	
10	*K.pneumoniae*	Blood	48	yes	vent	4.5	Yes
11	*K.pneumoniae*	Blood	83	yes	vent	5.5	Yes
12	*K.pneumoniae*	Blood	81	yes	vent	5.1	Yes
13	*K.pneumoniae*	Catheter	61	yes	vent	4.7	Yes
14	*K.pneumoniae*	Sputum	69	no	6liter	2.5	No
15	*K.pneumoniae*	Urine	69	no	6 liter	2.5	No
16	*K.pneumoniae*	Urine	44	no	8liter	1.9	No
17	*K.pneumoniae*	Wound	20	yes	2.3lite	1.9	
18	*K.pneumoniae*	Blood	38	no	4liter	2.5	No
19	*K.pneumoniae*	Sputum	68	yes	4liter	2.1	No
20	*K.pneumoniae*	Sputum	29	no	6 liter	2.1	No
21	*K.pneumoniae*	Wound	46	no	6liter	2.1	No
22	*K.pneumoniae*	Blood	43	no	4liter	2.4	No
23	*K.pneumoniae*	Blood	70	yes	vent	5.8	Yes
24	*K.pneumoniae*	Pleural F	69	no	vent	4.3	No
25	*K.pneumoniae*	Sputum	51	no	yes	3.1	No
26	*K.pneumoniae*	Wound	55	no	yes	4.6	No
27	*K.pneumoniae*	Sputum	36	no	6liter	2.2	No
28	*K.pneumoniae*	Urine	59	no			No

29	*K.pneumoniae*	Urine	53	yes		4.6	
30	*K.pneumoniae*	Wound	20	no	6liter	2.1	No
31	*K.pneumoniae*	Wound	20	no	vent	NA	No
32	*K.pneumoniae*	Blood	47	no	NA	NA	NA
33	*K.pneumoniae*	Sputum	47	no	NA	NA	NA
34	*K.pneumoniae*	Sputum	36	yes	6liter	2.1	NA
35	*K.pneumoniae*	Blood	45	no	6liter	2.1	No
36	*K.pneumoniae*	Wound	66	no	8liter	3.3	No
37	*K.pneumoniae*	Urine	70	yes	vent	3.5	Yes
38	*K.pneumoniae*	Urine	64	yes	NA	NA	No
39	*K.pneumoniae*	Sputum	64	yes	vent	NA	NA
40	*K.pneumoniae*	Wound	64	yes	NA	NA	NA
41	*K.pneumoniae*	Urine	41	yes	NA	NA	NA
42	*K.pneumoniae*	Urine	56	no	yes	3.9	No
43	*K.pneumoniae*	Urine	67	yes	yes	4.6	No
44	*K.pneumoniae*	Blood	71	no	yes	4.1	No
45	*K.pneumoniae*	Urine	85	yes	vent	3.6	Yes
46	*K.pneumoniae*	Screen	67	no	yes	4.5	No
47	*K.pneumoniae*	Urine	52	no	8iters	2.7	No
48	*K. pneumoniae*	Wound	75	no	8iters	2.1	No
49	*A.baumannii*	Wound	39	no	6 liter	1.6	No
50	*A.baumannii*	Blood	79	yes	vent	5.1	Yes
51	*A.baumannii*	Bloody sputm	95	yes	vent	6.2	Yes
52	*A.baumannii*	Blood	83	yes	vent	6.2	Yes
53	*A.baumannii*	Blood	70	yes	vent	5.5	Yes
54	*A.baumannii*	Wound	60	no	8liter	2.7	No
55	*A.baumannii*	Sputum	47	no	4 liter	2.5	No
56	*A.baumannii*	Sputum	38	yes	vent	5.9	Yes
57	*A.baumannii*	Blood	60	yes	vent	4.1	No
58	*A.baumannii*	Wound	33	no	8liter	2.2	No

59	*A.baumannii*	Sputum	69	no	4 liter	2.5	
60	*A.baumannii*	Blood	69	no	6 liter	2.2	No
61	*A.baumannii*	Blood	40	no	8liter	2.9	No
62	*A.baumannii*	Blood	59	yes	vent	3.7	Yes
63	*A.baumannii*	Sputum	69	no	4 liter	2.5	Yes
64	*A.baumannii*	Blood	67	no	4 liter	2.5	NA
65	*A.baumannii*	Blood	62	yes	vent	5.5	Yes
66	*A.baumannii*	Sputum	62	no	4 liter	2.5	NA
67	*A.baumannii*	Sputum	27	no	6liter	3.1	No
68	*A.baumannii*	Blood	73	no	yes	4.6	No
69	*A.baumannii*	Sputum	84	yes	vent	5.1	Yes
70	*A.baumannii*	Blood	47	no	4 liter	2.5	Yes
71	*A. baumannii*	Sputum	65	yes	vent	4.3	Yes
72	*A.baumannii*	Pleural F	34	no	6liter	1.9	No
73	*A.baumannii*	Sputum	57	no	yes	4.4	No
74	*A. baumannii*	Blood	73	no	4 liter	2.5	No
75	*A. baumannii*	Sputum	84	no	4 liter	2.5	NA
76	*A. baumannii*	Blood	54	no	8liter	3.3	No
77	*A. baumannii*	Blood	36	no	4 liter	2.5	
78	*A. baumannii*	Wound	57	no	yes	3.7	No
79	*A. baumannii*	Blood	51	no	yes	4.4	No
80	*A. baumannii*	Sputum	51	no	4 liter	2.5	
81	*A. baumannii*	Sputum	66	no	yes	3.7	No
82	*A. baumannii*	Sputum	55	no	8liter	3.4	No
83	*E.coli*	Blood	82	no	4 liter	2.5	
84	*E.coli*	Urine	61	yes	vent	5.3	Yes
85	*E.coli*	Sputum	63	no	6liter	2.7	No
86	*E.coli*	Urine	81	yes	vent	4.9	Yes
87	*E.coli*	Urine	74	no	yes	4.7	No
88	*E.coli*	Urine	76	no	yes	3.8	No

89	*E. coli*	Screen	58	no	yes	3.9	No
90	*E.coli*	Wound	72	no	yes	4.4	No
91	*E.coli*	Urine	57				No
92	*E.coli*	Blood	41	no	4liter	1.9	No
93	*E.coli*	Urine	45	no	8liter	2.7	No
94	*E.coli*	Urine	72	no	yes	3.7	No
95	*E.coli*	Urine	35	no	6liter	2.2	No
96	*E.coli*	Urine	34	no	8liter	1.9	No
97	*E.coli*	Urine	78	no	6iters	2.7	No
98	*E.coli*	Urine	62	no	yes	3.9	No
99	*E.coli*	Blood	69	no	yes	3.8	No
100	*E.coli*	Urine	49	no	6liter	2.8	No
101	*E.coli*	Urine	77	no	yes	3.8	No
102	*E.coli*	Urine	10			3.8	
103	*E.coli*	Wound	67	no	4 liter	1.2	No
104	*E.coli*	Urine	89	no	6 liter	1.4	No
105	*E.coli*	Wound	25	no	8iters	2.4	No
106	*E.coli*	Urine	83	yes	vent	4.2	Yes
107	*P.aeruginosa*	Sputum	62	no	6 liter	2.2	No
108	*P.aeruginosa*	Wound	60	no	8liter	1.9	No
109	*P.aeruginosa*	Sputum	76	no	8liter	2.4	No
110	*P.aeruginosa*	Wound	60	yes	yes	4.3	No
111	*P.aeruginosa*	Sputum	64	no	yes	3.7	No
112	*P.aeruginosa*	Wound	43	no	yes	4.3	No
113	*P.aeruginosa*	Wound	56	no	4liter	2.5	No
114	*P.aeruginosa*	Wound	53	no			No
115	*P.aeruginosa*	Sputum	68	no	8liter	2.5	No
116	*P.aeruginosa*	Sputum	81	yes	vent	5.1	Yes
117	*P.aeruginosa*	Wound	37	no	6liter	2.4	No
118	*Providentia*	Wound	43	no	6liter	2.3	No

119	*Enterobacter*	Urine	52	no	vent	2.7	No
120	*Eneterobacter*	Wound	66	no	yes	4.1	No
121	*Providencia*	Wound	72	no	8liter	3.3	No
122	*Providencia*	Wound	40	no	6liter	3.1	No
123	*Serratia*	Sputum	58				Yes
124	*Serratia*	sputum	73	yes	vent	3.1	Yes
125	*morganella*	Pleural F	65	yes	vent	4.4	Yes
126	*morganella*	Urine	79	yes	vent	3.6	Yes
127	*Stenotrophomonas*	Blood	45	no	6liter	3.3	No
128	*Achromobacte*	Urine	56	no	4liter	2.2	No
129	*C. koseri*	Urine	82	yes	vent	6.1	Yes

*Specimens were color coded for easy access. $^{+}$Age range color: 1 to 20 = light green, 21 to 49 dark green, over 50 blue. CXR = chest x-ray. h-O_2 = highest oxygen requirement during admission. HTN =hypertension. LALC =Lowest absolute lymphocyte count. CKD = chronic kidney disease. HF = heart failure. IHD = ischemic heart disease
